# A paralog of a bacteriochlorophyll biosynthesis enzyme catalyzes the formation of 1,2-dihydrocarotenoids in green sulfur bacteria

**DOI:** 10.1074/jbc.RA118.004672

**Published:** 2018-08-20

**Authors:** Daniel P. Canniffe, Jennifer L. Thweatt, Aline Gomez Maqueo Chew, C. Neil Hunter, Donald A. Bryant

**Affiliations:** From the ‡ Department of Molecular Biology & Biotechnology, University of Sheffield, Sheffield S10 2TN, United Kingdom,; the § Department of Biochemistry & Molecular Biology, The Pennsylvania State University, University Park, Pennsylvania 16802, and; the ¶ Department of Chemistry & Biochemistry, Montana State University, Bozeman, Montana 59717

**Keywords:** photosynthesis, photosynthetic pigment, carotenoid, bacterial genetics, energy metabolism, Blastochloris viridis, Chlorobaculum tepidum, green sulfur bacterium, Rhodobacter sphaeroides

## Abstract

*Chlorobaculum tepidum*, a green sulfur bacterium, utilizes chlorobactene as its major carotenoid, and this organism also accumulates a reduced form of this monocyclic pigment, 1′,2′-dihydrochlorobactene. The protein catalyzing this reduction is the last unidentified enzyme in the biosynthetic pathways for all of the green sulfur bacterial pigments used for photosynthesis. The genome of *C. tepidum* contains two paralogous genes encoding members of the FixC family of flavoproteins: *bchP*, which has been shown to encode an enzyme of bacteriochlorophyll biosynthesis; and *bchO*, for which a function has not been assigned. Here we demonstrate that a *bchO* mutant is unable to synthesize 1′,2′-dihydrochlorobactene, and when *bchO* is heterologously expressed in a neurosporene-producing mutant of the purple bacterium, *Rhodobacter sphaeroides*, the encoded protein is able to catalyze the formation of 1,2-dihydroneurosporene, the major carotenoid of the only other organism reported to synthesize 1,2-dihydrocarotenoids, *Blastochloris viridis*. Identification of this enzyme completes the pathways for the synthesis of photosynthetic pigments in *Chlorobiaceae*, and accordingly and consistent with its role in carotenoid biosynthesis, we propose to rename the gene *cruI*. Notably, the absence of *cruI* in *B. viridis* indicates that a second 1,2-carotenoid reductase, which is structurally unrelated to CruI (BchO), must exist in nature. The evolution of this carotenoid reductase in green sulfur bacteria is discussed herein.

## Introduction

Carotenoids are ubiquitous pigments of photosynthesis, and together with the modified tetrapyrrole molecules chlorophyll (Chl)^4^ and/or bacteriochlorophyll (BChl), are found in all naturally occurring Chl-dependent phototrophs (*i.e.* chlorophototrophs) (1) discovered to date. These isoprenoid molecules are used to harvest light by absorption of wavelengths in the 400–550-nm range of the solar spectrum and subsequently transfer excitation energy to (B)Chls in photochemical reaction center (RC) complexes where charge separation occurs (2). Carotenoids also play roles in photoprotection (via quenching of (B)Chl triplet states and scavenging of harmful radicals and singlet oxygen) and the stabilization of pigment-protein interactions in photosynthetic complexes of chlorophototrophic prokaryotes and plants (2–4). Carotenoids can also be synthesized by nonphototrophic organisms, including bacteria, fungi, and, surprisingly, insects (5). Remarkably, more than 1100 variants of these usually C_40_ isoprenoid compounds have been described thus far (6).

**Figure 1.**
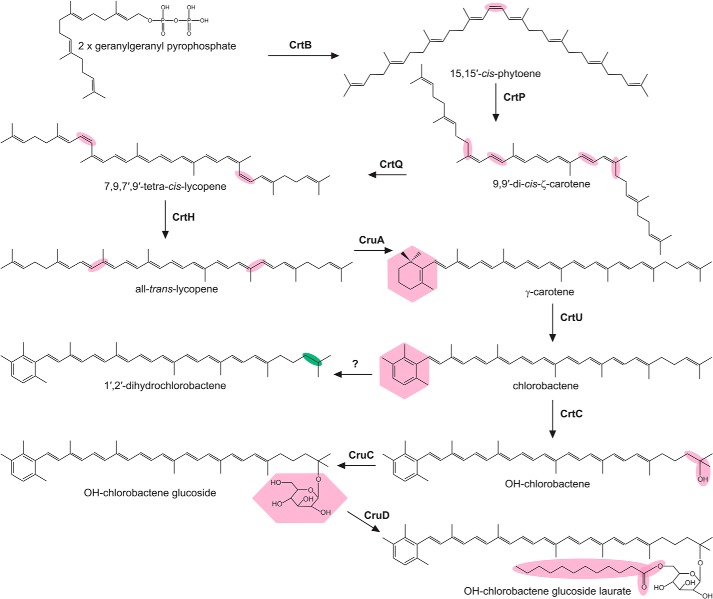
**Simplified carotenoid biosynthesis pathway in *C. tepidum*.** Enzymes catalyzing known steps are next to *arrows*, and respective modifications are highlighted in *pink*. Some enzymes can modify multiple substrates, *e.g.* OH-γ-carotene glucoside laurate can be formed by sequential modifications to γ-carotene by CrtC, CruC, and CruD, respectively. The enzyme catalyzing the formation of 1′,2′-dihydrochlorobactene is unknown; the modification introduced by this enzyme is highlighted in *green*.

The phototrophic green sulfur bacteria (GSB) are major primary producers of biomass in anoxic environments and contribute significantly to the biogeochemical cycling of carbon, nitrogen, and sulfur on Earth (7). They are anoxygenic chlorophototrophs that support photosynthesis at extremely low irradiance by using specialized light-harvesting structures, chlorosomes, in which BChls *c*, *d*, or *e* molecules self-aggregate to form highly efficient yet elegantly simple antenna complexes (8). The major carotenoids found in GSB have cyclic, aromatic end groups (9, 10), and >90% of the carotenoids found in *Chlorobaculum tepidum* are located in the interior of the chlorosome (11, 12). GSB species with chlorosomes composed of BChl *c* (such as *C. tepidum*) or BChl *d* (such as *Chlorobaculum parvum*) mostly produce a monocyclic aromatic carotenoid, chlorobactene. Brown-colored GSB species, such as *Chlorobaculum limnaeum*, which synthesize BChl *e* as their main antenna BChl and can grow at greater depths in stratified lakes, primarily make the dicyclic carotenoids isorenieratene and/or β-isorenieratene (7). Using a combination of genetic manipulation and heterologous expression approaches, the majority of the enzymes involved in carotenoid biosynthesis in GSB have been identified (13–16). The carotenoid biosynthesis pathway in *C. tepidum* is summarized in Fig. 1, and the structures of the major carotenoids of *C. limnaeum* are shown in Fig. S1. Only the enzyme catalyzing the reduction of chlorobactene to produce 1′,2′-dihydrochlorobactene in *C. tepidum* remained to be identified. The only other organism reported to synthesize 1,2-dihydrocarotenoids is the purple bacterium *Blastochloris viridis*, which contains 15,15′-*cis*-1,2-dihydroneurosporene in its RC (17).

In this study we identify the 1′,2′-carotenoid reductase responsible for the synthesis of dihydrocarotenoids in *C. tepidum* via mutation and heterologous expression in a purple bacterial host. The identification of this gene completes the biosynthetic pathways for the carotenoids, and, together with the recent completion of the pathways for (B)Chls in GSB (18, 19), the pathways for the synthesis of all of the photopigments found in chlorophototrophic members of this phylum are now complete. Furthermore, bioinformatic analyses of the genome of *B. viridis*, and subsequent genetic manipulations reveal that a second, independently evolved carotenoid 1,2-reductase must exist in nature.

## Results

### Disruption of bchO prevents the synthesis of 1′,2′-dihydrochlorobactene in C. tepidum

**Figure 2.**
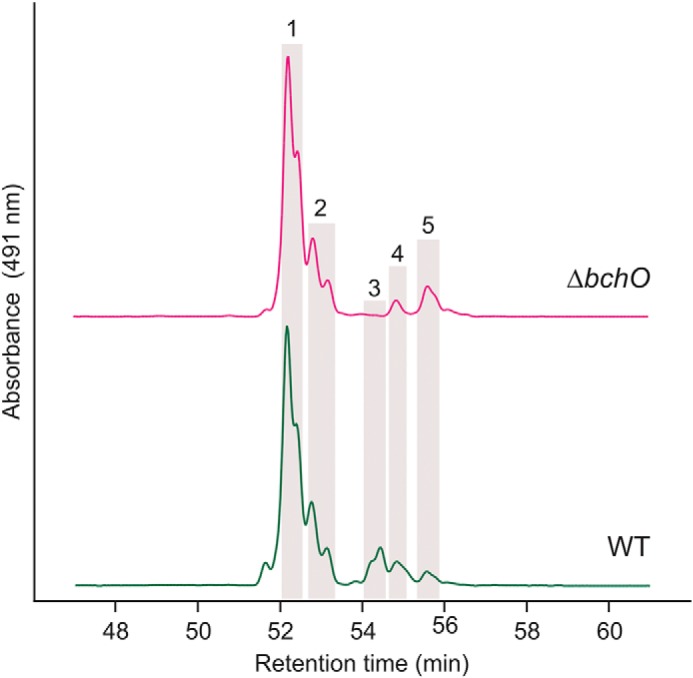
**HPLC elution profiles of carotenoids extracted from *C. tepidum* strains.** Highlighted peaks indicate the following carotenoids: *peak 1*, chlorobactene; *peak 2*, OH-chlorobactene glucoside laurate; *peak 3*, 1′,2′-dihydrochlorobactene; *peak 4*, OH-γ-carotene glucoside laurate; and *peak 5*, γ-carotene. Carotenoids are identified as in Ref. 13, and two peaks for each pigment are present because of the existence of *trans*- and *cis-*conformations of each.

A previous study demonstrated that, of the two paralogs of the BChl biosynthesis gene, *bchP*, ORF CT2256 encodes an active BchP enzyme, whereas the role of the protein encoded by CT1232 (annotated as *bchO*) could not be established (20). The BchP and BchO proteins group with the FixC superfamily of electron-transfer, flavin-dependent reductases (21) and are members of the larger NAD(P)-binding Rossmann fold superfamily. BchP sequentially reduces three C=C bonds of the isoprenoid alcohol attached to a bacteriochlorin macrocycle, and ChlP performs the same function in the biosynthesis of Chls, converting the geranylgeranyl moiety to phytyl (22) (Fig. S2). C_40_ carotenoids are synthesized from two molecules of the isoprenoid compound, geranylgeranyl pyrophosphate, and thus the reaction to reduce the 1,2 (or 1′,2′) C=C bond, such as that carried by 1′,2′-dihydrochlorobactene in *C. tepidum*, is reminiscent of the reduction reactions catalyzed by BchP. To determine whether *bchO* encodes a carotenoid 1,2-reductase, the CT1232 ORF was interrupted by insertional inactivation with an *aadA* cassette that confers resistance to spectinomycin; the interruption was confirmed by colony PCR (Fig. S3) and subsequent sequencing of the DNA amplicon. The resulting strain, Δ*bchO*, was grown in liquid medium, the accumulated pigments were extracted, and, along with those from the WT, were analyzed by HPLC (Fig. 2). As expected, 1′,2′-dihydrochlorobactene was detected in the WT. Disruption of *bchO* abolished the production of 1′,2′-dihydrochlorobactene, indicating that BchO plays a role in the reduction of the 1′,2′ double bond in carotenoids of *C. tepidum*. The synthesis of all other carotenoids in *C. tepidum* was unaffected.

### Expression of certain GSB bchO genes in Rhodobacter sphaeroides results in the accumulation of 1,2-dihydrocarotenoids

**Figure 3.**
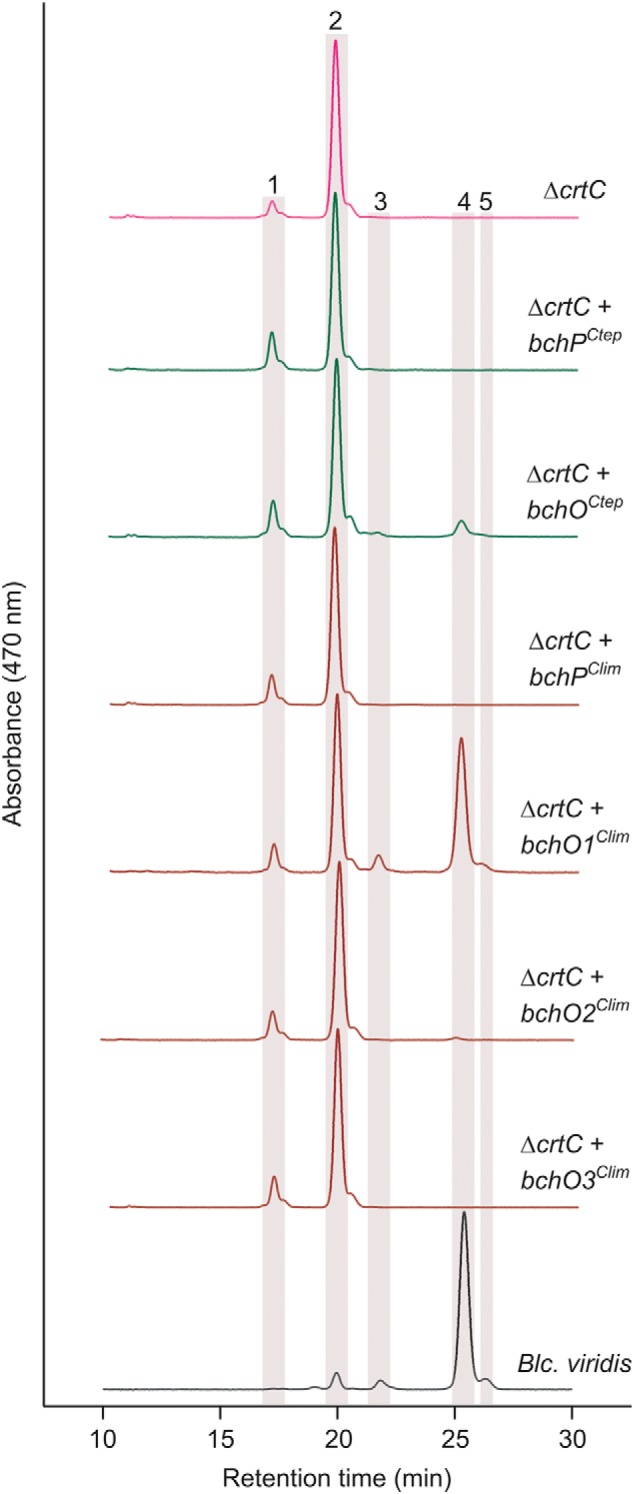
**HPLC elution profiles of carotenoids extracted from *R. sphaeroides* Δ*crtC* strains expressing GSB *bchP* and *bchO* paralogs.** Carotenoids extracted from *B. viridis* are included for comparison. Highlighted peaks indicate the following carotenoids: *peak 1*, lycopene; *peak 2*, neurosporene; *peak 3*, 1,2-dihydrolycopene; *peak 4*, 1,2-dihydroneurosporene; and *peak 5*, 15,15′-*cis*-1,2-dihydroneurosporene.

The purple phototrophic bacterium *B. viridis* is the only organism other than *C. tepidum* that has been documented to produce 1,2-dihydrocarotenoids, utilizing 1,2-dihydroneurosporene as its major carotenoid (23). The 15,15′-*cis* isomer of this carotenoid is found in the RC, and 1,2-dihydrolycopene is also detected as a minor pigment (24) (Fig. S4). The presumptive precursor for the major carotenoid of *B. viridis*, neurosporene, accumulates to high levels in a Δ*crtC* mutant of the model purple phototrophic bacterium, *R. sphaeroides* (25). This mutant can serve as a platform in which to test whether the gene products of GSB *bchO* genes are sufficient to reduce the 1,2 C=C bonds of a carotenoid, by using pigments extracted from *B. viridis* as standards for the product(s) of the reaction. The *bchP* and *bchO* genes from *C. tepidum* (*bchP^Ctep^* and *bchO^Ctep^*), along with *bchP* and the three *bchO* paralogs found in the genome of the brown-colored, BChl *e*-producing *C. limnaeum* (*bchP^Clim^* and *bchO1-3^Clim^*, where *bchO1^Clim^* encodes a protein most similar to BchO*^Ctep^*), were cloned into the pBBRBB–P*puf*_843–1200_ plasmid (26), in which transcription is controlled by the promoter found upstream of the genes encoding the core light-harvesting antenna (LH1) and RC subunits in *R. sphaeroides* (27). These plasmids were conjugated into the Δ*crtC* mutant of *R. sphaeroides*, the resulting strains were grown in liquid medium, and carotenoids were extracted and analyzed by HPLC (Fig. 3). As expected, the Δ*crtC* mutant primarily accumulated neurosporene, as well as a smaller amount of lycopene. The strains expressing *bchP^Ctep^* and *bchP^Clim^* made the same carotenoids as the Δ*crtC* mutant. The strains expressing *bchO^Ctep^* and *bchO2^Clim^* made small amounts of 1,2-dihydroneurosporene, the level in the latter strain being just above the limit of detection. The strain expressing *bchO1^Clim^* accumulated 1,2-dihydroneurosporene at close to the same level as neurosporene, and both 15,15′-*cis*-1,2-dihydroneurosporene and 1,2-dihydrolycopene were also detected (Fig. 3). Dihydrocarotenoids were not detected in the strain expressing *bchO3^Clim^*.

**Figure 4.**
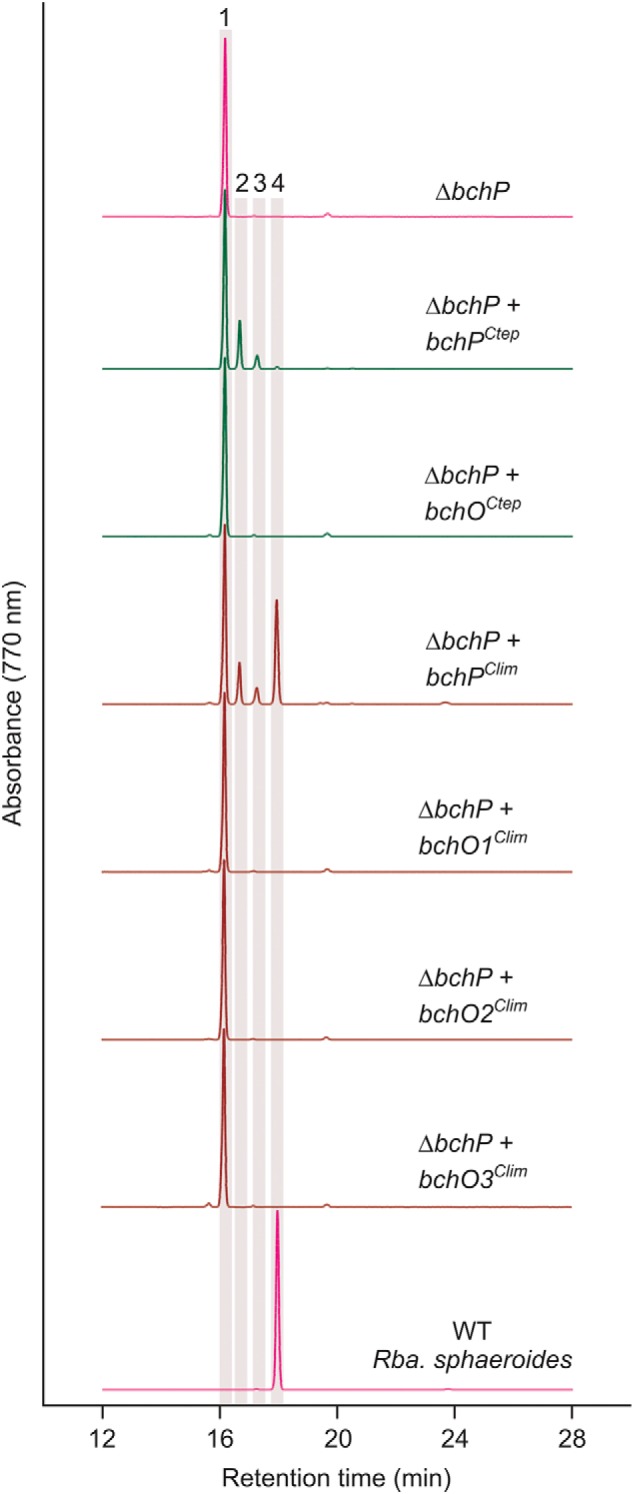
**HPLC elution profiles of BChl species extracted from *R. sphaeroides* Δ*bchP* strains expressing GSB *bchP* and *bchO* paralogs.** BChl *a* extracted from WT *R. sphaeroides* is included for comparison. Highlighted peaks indicate BChl *a* species esterified with the following isoprenoid alcohols: *peak 1*, geranylgeraniol; *peak 2*, dihydrogeranylgeraniol; *peak 3*, tetrahydrogeranylgeraniol; and *peak 4*, phytol.

To confirm the activities of these BchP proteins as BChl reductases and to test any potential activity of BchO on BChl, these plasmids were transformed into a Δ*bchP* mutant of *R. sphaeroides* (28) that accumulates BChl *a* carrying a polyunsaturated geranylgeraniol moiety (Fig. 4). Expression of GSB *bchP* genes in this background restored synthesis of BChl *a* esterified with phytol, although complete conversion to the mature pigment was not achieved; products with one and two reduced double bonds were also detected. The elution profiles from strains expressing *bchO* orthologs were identical to that of the Δ*bchP* mutant, indicating that these GSB genes do not encode enzymes able to reduce the alcohol moieties of BChl *a*. These results establish that BchO can act as a carotenoid 1,2-reductase and indicate that the enzyme is not involved in the biosynthesis of BChls. A conserved ORF found in the photosynthesis gene clusters of many purple phototrophic bacteria is also annotated as *bchO* (29), although the encoded proteins share no similarity to BchO of GSB. We therefore propose, according to Demerec nomenclatural rules, that the GSB *bchO* genes be renamed *cruI* to reflect their herein established role in carotenoid biosynthesis. We further propose to eliminate the use of *bchO* with respect to the paralogous ORFs in those GSB for which no function can currently be assigned. We suggest that only locus tags be used to identify those ORFs.

### Phylogenetic analysis of BchP, CruI, and paralogous proteins

**Figure 5.**
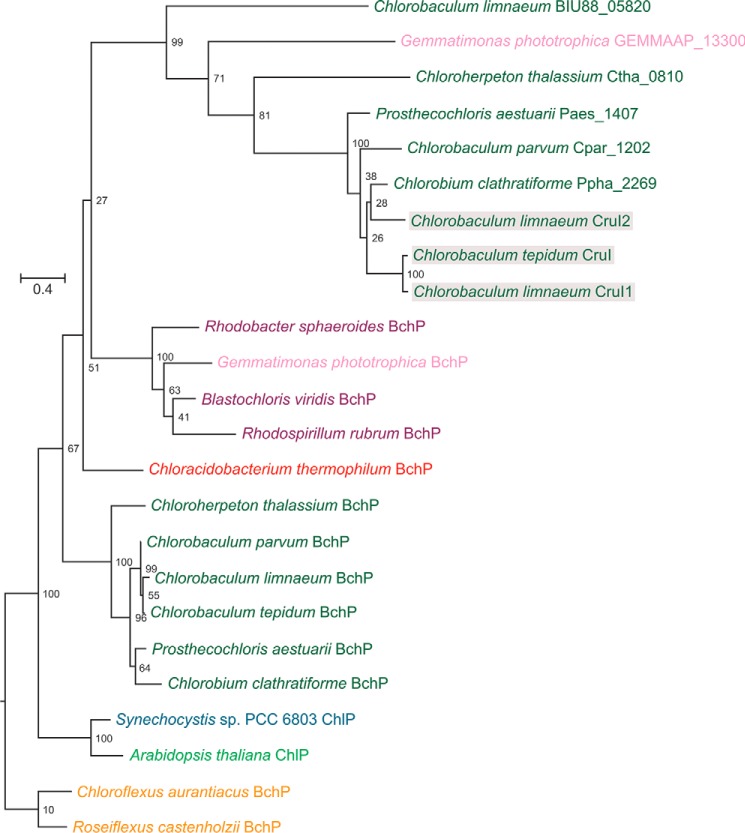
**Phylogenetic relationships of BchP/ChlP and CruI (BchO) paralogs.** The maximum likelihood tree was constructed from amino acid alignments using the PROTGAMMAAUTO model in RAxML version 8.2.4. The *numbers* on the branches indicate the percentage of bootstrap support from 100 replicates, and the *scale bars* indicate the specified number of amino acid substitutions per site. Example organisms from green sulfur bacteria (*green*), purple bacteria (*purple*), green filamentous bacteria (*Chloroflexi*; *amber*), *Acidobacteria* (*red*), and *Gemmatimonadetes* (*pink*) are included. Cyanobacterial (*cyan*) and plant (*light green*) ChlP proteins are included for reference. CruI proteins for which activity has been detected are *shaded* in *gray*.

To investigate the evolutionary relationship between BchP and CruI, orthologs of each were identified in the species listed in Table S1, through the use of the *C. tepidum* protein sequences as queries in Blastp searches of the respective proteomes. The phylogenetic relationships among BchP, CruI, and their paralogs are shown in Fig. 5. The tree supports the assertion that *cruI* is paralogous to *bchP* but that it may have arisen through horizontal gene transfer from a purple phototrophic bacterium, because the GSB CruIs are more closely related to purple bacterial BchPs than those within GSB. Interestingly, the only *cruI*-like gene identified in an organism other than GSB was found in *Gemmatimonas phototrophica*, a recently discovered member of the *Gemmatimonadetes*, the seventh bacterial phylum to contain a chlorophototroph (30). *G. phototrophica* acquired its phototrophic capability via horizontal gene transfer of the photosynthesis gene cluster from a purple chlorophototroph (31); the presence of this paralog in *G. phototrophica* raises interesting questions about its origin.

### R. sphaeroides strains expressing additional GSB cruI homologs do not synthesize 1,2-dihydrocarotenoids

The phylogenetic analysis detailed above identified homologs of *cruI* in additional GSB, although these genes are not completely conserved in this phylum. This suggested that other GSB may contain active carotenoid 1′,2′-reductases. To test this hypothesis, the identified ORFs (from *C. parvum*, *Chloroherpeton thalassium*, *Prosthecochloris aestuarii*, and *Chlorobium clathratiforme*) were subsequently tested in the *R. sphaeroides* Δ*crtC* mutant in the same manner described above. Additionally, an apparent *bchP* paralog present in the genome of *G. phototrophica* was also tested (Fig. S5). The expression of these *bchP*/*cruI* homologs in the Δ*crtC* mutant did not result in the production of 1,2-dihydroneurosporene. It may be that these proteins display stricter substrate specificity than those from *C. tepidum* and *C. limnaeum*, only catalyzing the reduction of chlorobactene, γ-carotene, or some other compound (*e.g.* Chl *a* or a lipid). However, 1,2-dihydrocarotenoids have not been reported in these GSB strains, and dihydrocarotenoids do not seem to be present in *G. phototrophica* (32).

### The B. viridis carotenoid 1,2-reductase is unrelated to CruI

**Figure 6.**
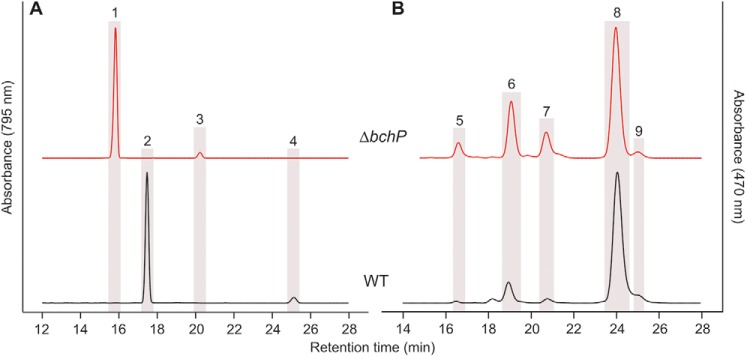
**HPLC elution profiles of BChls (*A*) and carotenoids (*B*) extracted from *B. viridis* strains.** Highlighted peaks indicate the following pigments: *peak 1*, BChl *b* esterified with geranylgeraniol; *peak 2*, BChl *b* esterified with phytol; *peak 3*, BPheo *b* esterified with geranylgeraniol; *peak 4*, BPheo *b* esterified with phytol; *peak 5*, lycopene; *peak 6*, neurosporene; *peak 7*, 1,2-dihydrolycopene; *peak 8*, 1,2-dihydroneurosporene; *peak 9*, 15,15′-*cis*-1,2-dihydroneurosporene.

The recently sequenced genome of *B. viridis*, the only strain outside the GSB documented to produce carotenoids reduced at the 1,2 position, does not contain *cruI* (33). In addition to the carotenoids depicted in Fig. S4, *B. viridis* utilizes BChl *b* as its primary photopigment (34) and bacteriopheophytin (BPheo) *b*, a demetallated analog of its parent BChl that acts as the primary electron acceptor in type-2 RCs (35). Both of these pigments carry a reduced phytyl moiety (36), and thus *B. viridis* must contain an active BchP. Because *cruI* is a paralog of *bchP* and catalyzes a similar reductive reaction, the *B. viridis bchP* (BVIR_564) gene was deleted to determine whether the encoded protein is a bifunctional BChl/carotenoid reductase. The gene was replaced at its start codon with the *aadA* gene, and this replacement was confirmed by colony PCR (Fig. S6) and subsequent sequencing of the DNA amplicon. The resulting strain, Δ*bchP*, was cultured in liquid medium, and the accumulated pigments were extracted and, along with those from the WT, analyzed by HPLC (Fig. 6). Deletion of *bchP* results in shifts in retention for both the major BChl *b* peak and the minor BPheo *b* peak to shorter times, consistent with the effect of mutations of *chlP*/*bchP* genes in organisms synthesizing Chl *a*/BChl *a* (37, 38) (Fig. 4). The BChl *b* and BPheo *b* in the Δ*bchP* mutant should now be esterified with geranylgeraniol rather than phytol, indicating that the activity of BchP has been abolished. The carotenoids from this strain were also analyzed; although the levels of lycopene and 1,2-dihydrolycopene are reduced in Δ*bchP* relative to the WT, both carotenoids were still detected. The major carotenoid in Δ*bchP* is 1,2-dihydroneurosporene. These results indicate that BchP in *B. viridis* is solely utilized for the synthesis of BChl *b* carrying a reduced esterifying alcohol moiety and that it is not responsible for the production of 1,2-dihydrocarotenoids in this organism.

## Discussion

Our identification of CruI as a 1,2-carotenoid reductase in *C. tepidum* completes the pathways for the biosynthesis of carotenoids in GSB, and thus the pathways for the synthesis of all photosynthetic pigments in the *Chlorobiaceae* (GSB) are now known. Orthologs of *cruI* are irregularly found throughout the GSB, although the detection of 1,2-dihydrocarotenoids has not been reported for any additional GSB species. It may be that these *cruI* genes are redundant in these species. *C. clathratiforme* is a brown-colored GSB, synthesizing BChl *e* as well as dicyclic carotenoids such as those depicted in Fig. S1; these carotenoids do not have ψ-end groups available for reduction, so redundancy of *cruI* from this strain is unsurprising. However, *C. limnaeum* also accumulates BChl *e* and dicyclic carotenoids (10), but we have demonstrated that this strain contains at least two active CruI carotenoid reductases. *C. parvum*, *C. thalassium* (*Chloroherpetonaceae*), and *P. aestuarii* are green-colored GSB and, like *C. tepidum*, synthesize monocyclic carotenoids with chlorobactene or γ-carotene backbones (39, 40), but the CruI proteins from these strains may have lost function during their evolution. Alternatively, the substrate provided to these proteins may be unsuitable to determine their activities as carotenoid reductases; they may have stricter substrate specificities and only reduce carotenoids with chlorobactene or γ-carotene backbones. It is also possible that the proteins encoded by these genes have alternative functions, *e.g.* biosynthesis of other isoprenoid molecules or the reduction/desaturation of fatty acids. The role of the CruI proteins for which an activity has not been demonstrated will require further study, including heterologous expression in the *cruI* mutant of *C. tepidum*, which may produce more suitable substrates for these orthologs. This will require the development of a plasmid-based expression system or the identification of a neutral site in the genome in which a foreign gene and a promoter sequence can be inserted.

The absence of a *cruI* gene in *B. viridis*, which synthesizes 1,2-dihydro-derivatives of neurosporene and lycopene, suggests that an additional carotenoid 1,2-reductase exists in nature, and it must be structurally unrelated to those found in GSB. This independent evolution of unrelated enzymes catalyzing the same reaction is not uncommon in nature (41) and is in fact quite prevalent in pathways for pigment biosynthesis; unrelated enzymes catalyzing three of the intermediate steps of (B)Chl biosynthesis are known to exist (42–45), and GSB and cyanobacteria utilize three or four enzymes to produce lycopene from phytoene, whereas purple phototrophs use a single phytoene desaturase enzyme (16, 46) (see Fig. 1 and Fig. S4).

Phylogenetic analysis of BchP and CruI paralogs suggested that GSB CruI proteins are more closely related to members of the purple bacterial BchP family than the BchP proteins within GSB. It could be that an ancestral GSB acquired a purple bacterial *bchP* gene that would have been redundant and that this gene accumulated mutations leading to its evolutionary conversion into a gene encoding a carotenoid reductase. Similarly, *G. phototrophica* acquired a purple bacterial photosynthesis gene cluster via lateral transfer and contains both *bchP* and a *cruI*-like gene; the *bchP* gene encodes a protein that groups with the purple bacterial BchP sequences as expected, but *cruI* genes are absent in purple bacteria. This raises the possibility that a gene duplication occurred in a purple bacterium, and *G. phototrophica* acquired two copies of *bchP*, one of which was also transferred to GSB at some point, or even that the duplication occurred in *G. phototrophica* and that *cruI* evolved in this phylum and was subsequently transferred to the GSB. It is possible that as more genome data are acquired, *cruI*-like genes will be identified in diverse phyla, such as the purple bacteria, which may clarify the evolutionary history of this enzyme.

The phylogenetic relationship between BchP and CruI proteins makes it of interest to elucidate the necessity for the evolution of carotenoid 1,2-reductases. 1′,2′-Dihydrochlorobactene is a minor carotenoid in *C. tepidum.* It only accounts for ∼6% of total carotenoids in the WT (13), and a null mutation of *cruI* has no detectable effect on growth (20). *C. limnaeum* contains two or possibly three active 1,2-reductases, even though it exclusively synthesizes carotenoids that cannot be reduced at these positions. Additionally, *cruI* genes are common in GSB genomes, but 1′,2′-dihydrocarotenoids have not been documented in these strains. It may be that 1′,2′-dihydrocarotenoids play an important role in light harvesting or quenching under as-yet-untested growth conditions in GSB, which may induce their synthesis in the strains that appear not to produce them under laboratory growth regimes. In *C. tepidum*, reduction of the 1′,2′ double bond would prevent hydroxylation by CrtC and thus prevent further modification, such as glucoside esterification (Fig. 1), which may be advantageous at irradiances analogous to those found deep in the water column. Similarly, in brown-colored GSB like *C. limnaeum*, the reduction of this bond may prevent cyclization at the ψ-end, resulting in the accumulation of monocyclic carotenoids; this method may be employed to tailor the absorption properties of the organism under specific growth conditions in which dicyclic carotenoids do not provide a growth advantage. We intend to explore this further in both green- and brown-colored GSB.

1,2-Dihydroneurosporene and 1,2-dihydrolycopene, found in *B. viridis*, have identical spectral properties to the common neurosporene and lycopene carotenoids found in purple phototrophic bacteria. This suggests that the necessity to reduce the 1,2 C=C bonds of these carotenoids may be structural rather than spectroscopic. Saturation of this bond would make the carotenoid more flexible at the reduced end, which may be required for assembly of the RC–LH1 complex; thus, loss of the gene encoding the carotenoid 1,2-reductase unrelated to CruI in the obligate phototroph *B. viridis* may be lethal. The native BChl *a* biosynthesis pathway of *R. sphaeroides* can be diverted toward the production of BChl *b* (47), and we have demonstrated that the expression of an active GSB *cruI* (in particular *cruI1^Clim^*) in a Δ*crtC* background of the same organism results in the production of the same complement of carotenoids produced by *B. viridis*. Thus, it may be possible to test the assembly of the *B. viridis* RC–LH1 complex in a strain of *R. sphaeroides* producing BChl *b* and dihydrocarotenoids and thereby determine the effect of the loss of a carotenoid 1,2-reductase in this background.

## Experimental procedures

### Growth conditions

Strains of GSB were grown in liquid CL medium or on solid CP medium as previously described (13, 48) and were incubated at 25 °C (or 42 °C for *C. tepidum*) under incandescent illumination (150 μmol photons·m^−2^·s^−1^). Cells for pigment analysis were grown in 25-ml cultures to early stationary phase before analysis. All strains and plasmids used in this study are listed in Table S2.

*R. sphaeroides* strains were grown under microoxic conditions in the dark in a rotary shaker at 30 °C in liquid M22+ medium (49) supplemented with 0.1% casamino acids, and kanamycin at 30 μg·ml^−1^ when required, with agitation at 150 rpm. *Escherichia coli* strains α-Select (Bioline) and S17-1 (50) transformed with plasmids described in the text were grown in a rotary shaker at 37 °C in LB medium supplemented with 30 μg·kanamycin ml^−1^.

*B. viridis* was grown phototrophically in anoxic sodium succinate medium 27 (N medium) (51) under incandescent illumination (100 μmol·photons·m^−2^·s^−1^) at 30 °C as previously described (47). When required, the medium was supplemented with kanamycin or spectinomycin at 30 μg·ml^−1^.

### Construction of a bchO mutant of C. tepidum

Sequences encompassing the upstream and 5′ end, the 3′ end and the downstream regions, of the CT1232 locus of *C. tepidum* were amplified with primer pair CT1232UpF and CT1232UpR and primer pair CT1232DownF and CT1232DownR, respectively. An *aadA* cassette, encoding aminoglycoside 3″-adenylyltransferase and conferring resistance to streptomycin and spectinomycin, was amplified together with the promoter region from pSRA81 (13) with primer pair CT1232aadAF and CT1232aadAR. The three resulting amplicons were fused by overlap extension PCR, and the linear DNA fragment was transformed into *C. tepidum* as previously described (52, 53). The resulting transformants were analyzed for complete segregation by PCR analysis and sequencing with primer pair CT1232UpF and CT1232DownR.

### Cloning of GSB bchP genes and their paralogs

GSB *bchP* genes and paralogous open reading frames were amplified from genomic DNA of described GSB strains (see text and Table S1) or from gBlocks synthesized by Integrated DNA Technologies, Inc. (Coralville, IA) with the relevant primer pairs (Table S3), digested with BglII/BamHI and SpeI, and ligated in place of the DsRed gene in pBBRBB–*Ppuf*_843–1200_–DsRed (26) digested with BglII and SpeI.

### Transformation of R. sphaeroides

Sequenced clones in the pBBRBB–*Ppuf*_843–1200_ vector backbone were conjugated into *R. sphaeroides* from *E. coli* S17-1, and transconjugants were selected on M22+ medium supplemented with kanamycin.

### Construction of a bchP mutant of B. viridis

Sequences ∼600 bp upstream and downstream of BVIR_564 were amplified with primer pair bchP^Bv^UpF and bchP^Bv^UpR and primer pair bchP^Bv^DownF and bchP^Bv^DownR, respectively. The resulting amplicons were fused by overlap extension PCR, digested with EcoRI and HindIII, and ligated into similarly digested pK18*mobsacB* (54). The *aadA* gene was amplified from pSRA81 with bchP^Bv^aadAF and bchP^Bv^aadAF, digested with NdeI and XbaI, and ligated between these sites in the overlap between the upstream and downstream regions of BVIR_564 in the pK18*mobsacB* construct, such that BVIR_564 would be precisely replaced with *aadA* between the corresponding start and stop codons. The resulting plasmid was verified by DNA sequencing and conjugated into *B. viridis* using a method previously described (55). Transconjugants in which the plasmid had integrated into the genome by homologous recombination were selected on N medium supplemented with kanamycin and spectinomycin (see above). A second recombination event was then promoted by *sacB*-mediated selection on N medium supplemented with 5% (w/v) sucrose, containing spectinomycin but lacking kanamycin. Sucrose- and spectinomycin-resistant, kanamycin-sensitive colonies had excised the allelic exchange vector through the second recombination event, and replacement of BVIR_564 with *aadA* was confirmed by colony PCR and sequencing using bchP^Bv^CheckF and bchP^Bv^CheckR primers.

### Extraction of pigments

Pigments were extracted from cell pellets with 7:2 acetone/methanol (v/v) as previously described (56). Carotenoids were further processed by addition of a drop of 5 m NaCl and an equal volume of hexane to the clarified acetone/methanol extract. The sample was mixed, and the phases were allowed to separate (57). The upper hexane phase was transferred to a glass vial, dried in a vacuum concentrator at 30 °C, and reconstituted in a small volume of 0.2% (v/v) ammonia in methanol (for analysis of carotenoids in GSB) or acetonitrile (for analysis of carotenoids in *R. sphaeroides* or *B. viridis*) prior to analysis by reversed-phase, HPLC.

### Analysis of pigments by reversed-phase HPLC

Pigments were separated at a flow rate of 1 ml·min^−1^ at room temperature on a Supelco Discovery HS C18 (5-μm particle size, 120 Å pore size, 250 × 4.6 mm) on an Agilent 1100 HPLC system. BChl *b* species were separated using a method modified from that of Ortega-Ramos *et al.* (28). Solvents A and B were 64:16:20 (v/v/v) methanol/acetone/H_2_O and 80:20 (v/v) methanol/acetone, respectively. Pigments were eluted with a linear gradient of 50–100% solvent B over 10 min, followed by further elution with 100% solvent B for 25 min. Elution of species of BChl *b* was monitored by checking the absorbance at 795 nm.

Carotenoids extracted from *C. tepidum* were separated and identified as previously described (13). Solvents A and B were 42:33:25 (v/v/v) methanol/acetonitrile/H_2_O and 50:20:30 (v/v/v) methanol/acetonitrile/ethyl acetate, respectively. Pigments were eluted with a linear gradient of 30% to 100% solvent B over 52 min, followed by further elution with 100% solvent B for 6 min. Elution of carotenoid species was monitored by monitoring the absorbance at 490 nm.

Carotenoids extracted from *R. sphaeroides* and *B. viridis* were separated using a method modified from that of Magdaong *et al.* (58). Pigments were eluted on an isocratic gradient of 58:35:7 (v/v/v) acetonitrile/methanol/THF. Elution of carotenoid species was monitored at 470 and 505 nm.

### Phylogenetic analysis of BchP paralogs

BchP and paralogous protein sequences from six GSB, three purple bacteria, two green filamentous bacteria (*Chloroflexi*), one acidobacterium (*Chloracidobacterium thermophilum*), and one member of the *Gemmatimonadetes* were used, with ChlP sequences from one higher plant and one cyanobacterium used as outgroup members (Table S1). The obtained amino acid sequences were aligned using MUSCLE (59) with default settings, and phylogenies were obtained with RAxML (60) version 8.2.4, using the automated protein model assignment algorithm and a gamma model of rate heterogeneity (-m PROTGAMMAAUTO).

## Author contributions

D. P. C. and D. A. B. conceptualization; D. P. C., J. L. T., A. G. M. C., C. N. H., and D. A. B. resources; D. P. C. and D. A. B. formal analysis; D. P. C., C. N. H., and D. A. B. funding acquisition; D. P. C. validation; D. P. C. visualization; D. P. C. and A. G. M. C. methodology; D. P. C. writing-original draft; J. L. T. and A. G. M. C. investigation; J. L. T., A. G. M. C., C. N. H., and D. A. B. writing-review and editing; D. A. B. supervision; D. A. B. project administration.

## Supplementary Material

Supporting Information

## Footnotes

4 The abbreviations used are:

Chl: chlorophyll
BChl: bacteriochlorophyll
RC: reaction center
GSB: green sulfur bacteria
BPheo: bacteriopheophytin.
