# Supplementary material for: A paralog of a bacteriochlorophyll biosynthesis enzyme catalyzes the formation of 1,2-dihydrocarotenoids in green sulfur bacteria
Source: J Biol Chem. 2018 Aug 20;293(39):15233–42. doi: 10.1074/jbc.RA118.004672 (PMC6166724; doi:10.1074/jbc.RA118.004672)
Supplement: Supporting Information [file supp_293_39_15233__index.html]

A paralog of a bacteriochlorophyll biosynthesis enzyme catalyzes the formation of 1,2-dihydro-carotenoids in green sulfur bacteria — Dihydro-carotenoid synthesis in Chlorobi — A paralog of a bacteriochlorophyll biosynthesis enzyme catalyzes the formation of 1,2-dihydrocarotenoids in green sulfur bacteria — Dihydrocarotenoid synthesis in Chlorobi — Supporting Information 

# A paralog of a bacteriochlorophyll biosynthesis enzyme catalyzes the formation of 1,2-dihydrocarotenoids in green sulfur bacteria

## Supporting Information

- Supporting Information (to be published online) - Supporting Information: Figures S1-S6 and Tables S1 to S3
