# Supplementary material for: A paralog of a bacteriochlorophyll biosynthesis enzyme catalyzes the formation of 1,2-dihydrocarotenoids in green sulfur bacteria
Source: J Biol Chem. 2018 Aug 20;293(39):15233–42. doi: 10.1074/jbc.RA118.004672 (PMC6166724; doi:10.1074/jbc.RA118.004672)
Supplement: Supporting Information [file supp_RA118.004672_139143_1_supp_186791_pdm468.pdf]

SUPPORTING INFORMATION

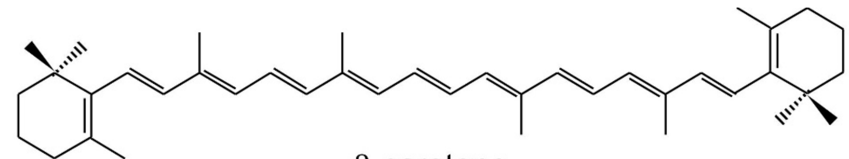

β-carotene

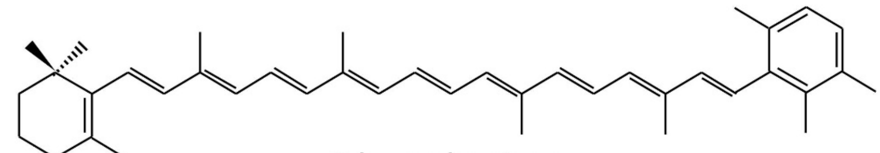

β-isorenieratene

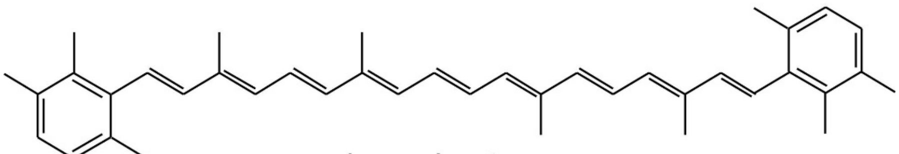

isorenieratene

Fig. S1. Major dicyclic carotenoids of *Cba. limnaeum* (and other brown-colored GSB).

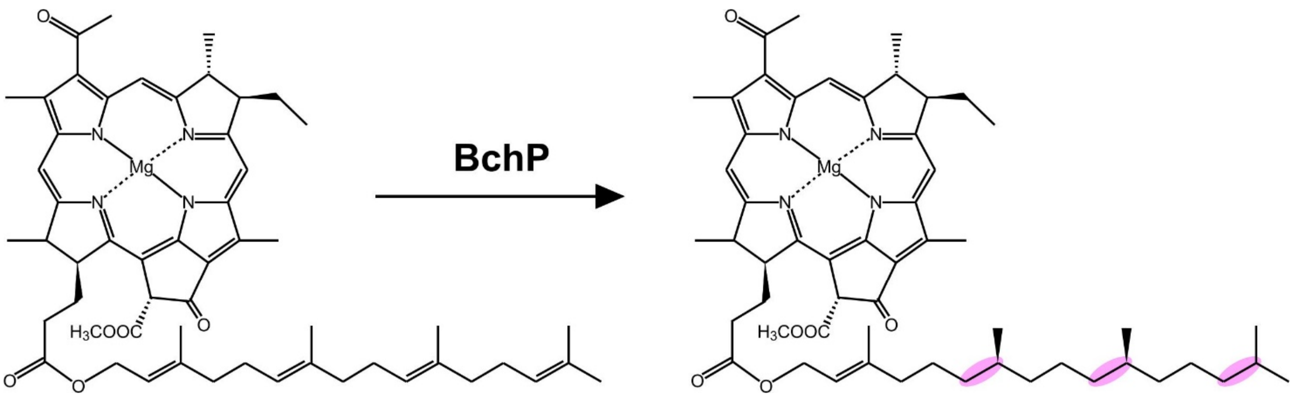

Fig. S2. Reaction catalyzed by BchP. The enzyme sequentially reduces three double bonds on the isoprenoid tail attached to a bacteriochlorin. ChlP catalyzes the same reaction on a Chl molecule. The figure depicts the conversion of BChl a_{GG} to BChl a_{P}. $_{GG}$; geranylgeraniol, $_{P}$; phytol.

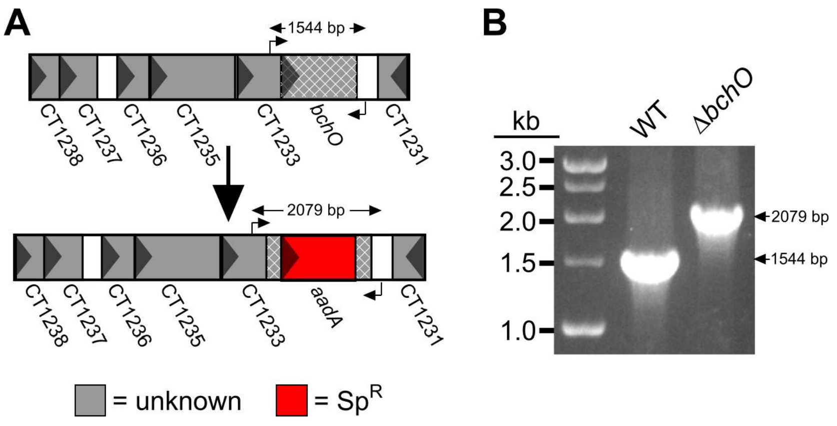

Fig. S3. Construction of a *Cba. tepidum bchO* mutant. *A*, diagram depicting the interruption of *bchO* with a spectinomycin resistance cassette, encoding the *aadA* gene. *B*, agarose gel electrophoresis of amplicons produced by colony PCR that confirm the replacement of *bchO* by *aadA*.

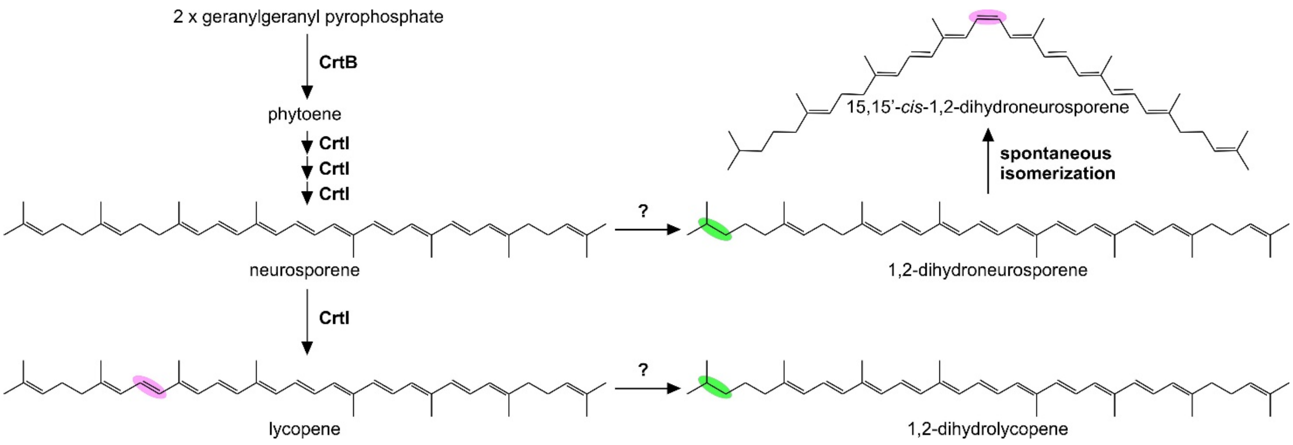

Fig. S4. Carotenoid biosynthesis pathway of *Blc. viridis*. Structures of carotenoids detected *in vivo* are shown. Enzymes catalyzing known steps are next to arrows, and respective modifications are highlighted in pink. The enzyme catalyzing the formation of 1,2-dihydro-neurosporene/lycopene is unknown; the modification carried by these carotenoids is highlighted in green.

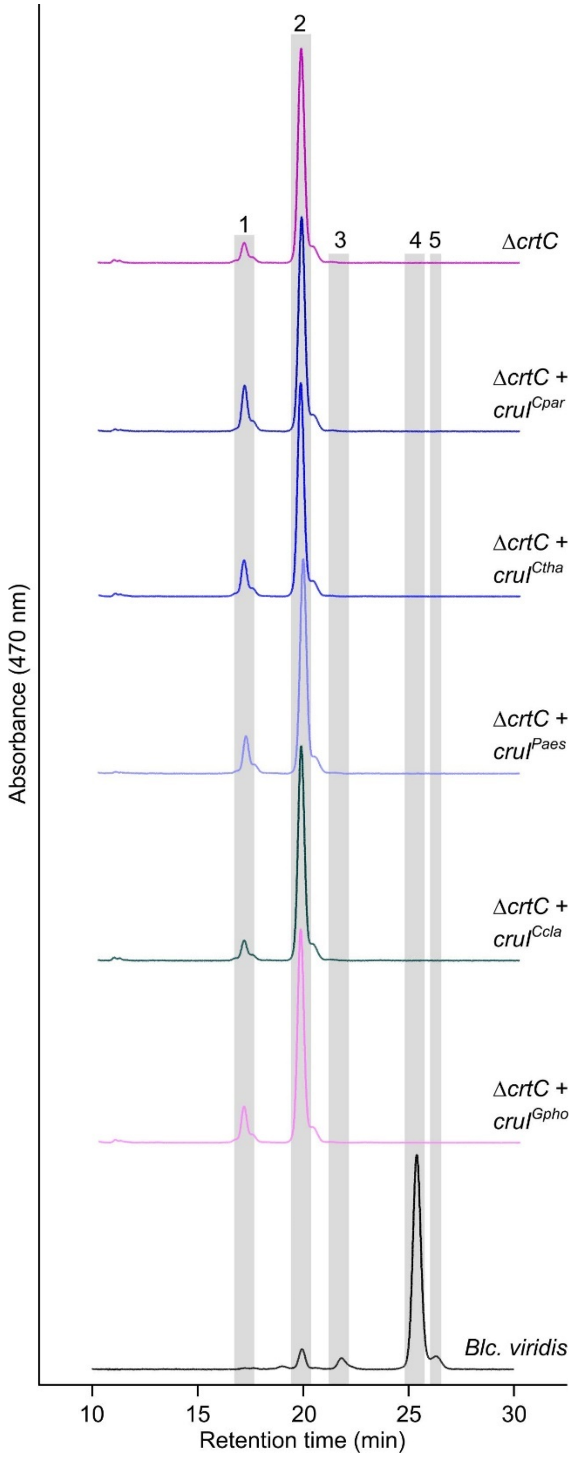

Fig. S5. HPLC elution profiles of carotenoids extracted from *Rba. sphaeroides* Δ*crtC* strains expressing additional GSB *cruI* paralogs. Carotenoids extracted from *Blc. viridis* are included for comparison. Highlighted peaks indicate the following carotenoids: 1; lycopene, 2; neurosporene, 3; 1,2-dihydrolycopene, 4; 1,2-dihydroneurosporene, 5; 15,15′-*cis*-1,2-dihydroneurosporene.

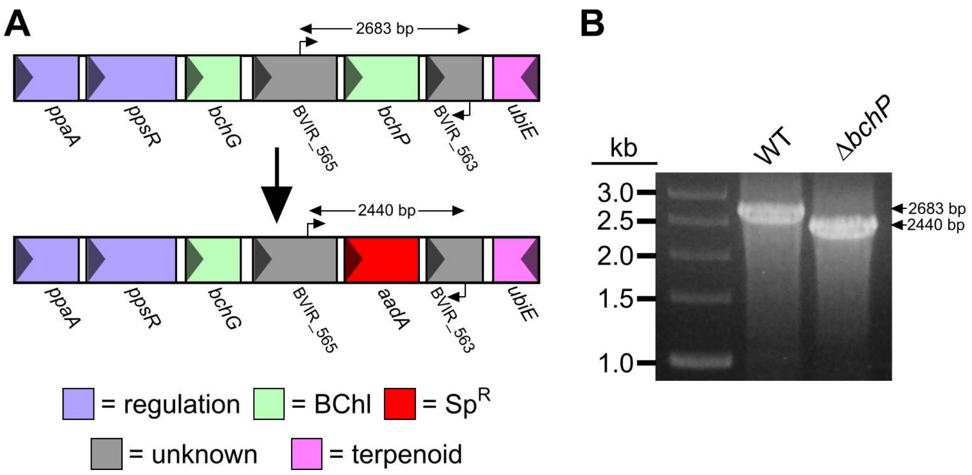

Fig. S6. Construction of a *Blc. virids bchP* mutant. *A*, diagram depicting the replacement of *bchP* with the spectinomycin resistance-conferring *aadA* gene. *B*, confirmation by colony PCR.

Protein	Accession Number
Arabidopsis thaliana ChlP	NP_177587.1
Synechocystis sp. PCC 6803 ChlP	WP_010872959.1
Chloroflexus aurantiacus BchP	WP_012257953.1
Roseiflexus castenholzii BchP	WP_012122214.1
Chloroacidobacterium thermophilum BchP	WP_014098661.1
Rhodobacter sphaeroides BchP	WP_002720441.1
Rhodospirillum rubrum BchP	WP_011388387.1
Blastochloris viridis BchP	WP_055036332.1
Gemmatimonas phototrophica BchP	WP_053334447.1
Gemmatimonas phototrophica GEMMAAP_13300	WP_043579498.1
Chlorobaculum tepidum BchP	WP_010933906.1
Chlorobaculum tepidum CruI	WP_010932902.1
Chlorobaculum limnaeum BchP	WP_069810857.1
Chlorobaculum limnaeum CruI1	WP_069809244.1
Chlorobaculum limnaeum CruI2	WP_069809391.1
Chlorobaculum limnaeum BIU88_05820	WP_069809527.1
Chlorobaculum parvum BchP	WP_012501285.1
Chlorobaculum parvum Cpar_1202	WP_012502441.1
Chloroherpeton thalassium BchP	WP_012499818.1
Chloroherpeton thalassium Ctha_0810	WP_012499362.1
Prosthecochloris aestuarii BchP	WP_012504626.1
Prosthecochloris aestuarii Paes_1407	WP_012505962.1
Chlorobium clathratiforme BchP	WP_012506884.1
Chlorobium clathratiforme Ppha_2269	WP_012508940.1

Table S1. Accession numbers for proteins used for phylogenetic analysis.
Sequences are from higher plant (light green), cyanobacteria (cyan), green sulfur bacteria (green), purple bacteria (purple), green filamentous bacteria (*Chloroflexi*; amber), *Acidobacteria* (red), and *Gemmatimonadetes* (pink).

Strain/Plasmid	Genotype/characteristics	Source
E. coli		
α-Select	Cloning strain for plasmid constructs	Bioline
S17-1	Conjugation strain for pK18*mobsacB* constructs	[1]
Cba. tepidum		
WT	TLS	[2]
Δ*bchO*	*aadA* replacement of central portion of CT1232, Sp^R	This study
Cba. limnaeum		
WT	DSM-1677	J. Imhoff*
Rba. sphaeroides		
WT	2.4.1	S. Kaplan†
Δ*crtC*	Unmarked deletion mutant of RSP_0267	[3]
Δ*bchP*	Unmarked deletion mutant of RSP_0277	[4]
Blc. viridis		
WT	DSM-133	DSMZ
Δ*bchP*	*aadA* replacement of BVIR_564, Sp^R	This study
**Plasmid**		
pK18*mobsacB*	Allelic exchange vector, Km^R	J. Armitage‡
pK18[*bchP*^{Bv} KO]	Sequence replacing BVIR_564 with *aadA* cloned into pK18*mobsacB* EcoRI/HindIII	This study
pBBRBB-*Ppuf*	Expression vector carrying the *puf* promoter of *Rba. sphaeroides*, Km^R	[5]
pBB[*bchP*^{Ctep}]	*Cba. tepidum* CT2256 cloned into pBBRBB-*Ppuf* between BglII/SpeI	This study
pBB[*bchO*^{Ctep}]	*Cba. tepidum* CT1232 cloned into pBBRBB-*Ppuf* as above	This study
pBB[*bchP*^{Clim}]	*Cba. limnaeum* BIU88_11310 cloned into pBBRBB-*Ppuf* as above	This study
pBB[*bchO1*^{Clim}]	*Cba. limnaeum* BIU88_04840 cloned into pBBRBB-*Ppuf* as above	This study
pBB[*bchO2*^{Clim}]	*Cba. limnaeum* BIU88_05430 cloned into pBBRBB-*Ppuf* as above	This study
pBB[*bchO3*^{Clim}]	*Cba. limnaeum* BIU88_05820 cloned into pBBRBB-*Ppuf* as above	This study
pBB[*bchO*^{Cpar}]	*Cba. parvum* Cpar_1202 cloned into pBBRBB-*Ppuf* as above	This study
pBB[*bchO*^{Ctha}]	*Chp. thalassium* Ctha_0810 cloned into pBBRBB-*Ppuf* as above	This study
pBB[*bchO*^{Paes}]	*Ptc. aestuarii* Paes_1407 cloned into pBBRBB-*Ppuf* as above	This study
pBB[*bchO*^{Ccla}]	*Chl. clathratiforme* Ppha_2269 cloned into pBBRBB-*Ppuf* as above	This study
pBB[*bchO*^{Gpho}]	*Gem. phototrophica* GEMMAAP_13300 cloned into pBBRBB-*Ppuf* as above	This study

Table S2. List of strains and plasmids used in this study.

*University of Kiel, Germany

†Department of Microbiology & Molecular Genetics, University of Texas Medical School, U.S.A.

‡Department of Biochemistry, University of Oxford, U.K.

Primer	Sequence (5’-3’)	Restriction site
CT1232UpF	CTTATGCTGCTCTTCCGCAAAC	
CT1232UpR	GTTACCACCGCTGCGTTCGCCATTCCGCAAAATCCTG	
CT1232DownF	CAAGGTAGTCGGCAAATAATGTCGTCAAGGAAAACGCATTTGC	
CT1232DownR	TTGCCACATCACCGCCATC	
CT1232aadAF	GAACGCAGCGGTGGTAAC	
CT1232aadAR	CATTATTTGCCGACTACCTTG	
bchP^{Ctep} pBB F	CGGGATCCATGCTGTATGATGTCGCAATCATAGG	BamHI
bchP^{Ctep} pBB R	GCGACTAGTTTACGAACCCTTCGCGAGGTGG	SpeI
bchO^{Ctep} pBB F	ATAGATCTATGCAGCGTTACGATGCAGTG	BglII
bchO^{Ctep} pBB R	GCGACTAGTTCAATAAAACGGATTCAGCAG	SpeI
bchP^{Clim} pBB F	CGGGATCCATGCTGTACGATGTCGCAGTCATAGG	BamHI
bchP^{Clim} pBB R	GCGACTAGTTCAACTTCCTTTTGCGAGGTGATAGACG	SpeI
bchO1^{Clim} pBB F	CGGGATCCATGCAGCGCTATGATGTGGTG	BamHI
bchO1^{Clim} pBB R	GCGACTAGTTCAATAAAACGGATTCAGCAGTTTG	SpeI
bchO2^{Clim} pBB F	CGGGATCCATGATATCTTATGACGCAGTGATTTCGG	BamHI
bchO2^{Clim} pBB R	GCGACTAGTTCAAAAAAACGGAACGATCAGTTTAAG	SpeI
bchO3^{Clim} pBB F	CGGGATCCATGAAAAACCACTACACGGCGGTTG	BamHI
bchO3^{Clim} pBB R	GCGACTAGTCTACGTTCCGGTGATCTTGTCGG	SpeI
bchP^{Bv}UpF	CCGGAATTCCCGTTCCGCGTCGCGCTTGCC	EcoRI
bchP^{Bv}UpR	GCAGGCGGCCGCGACGGATCGG*TCTAGA*TGCATGCATATGCTCATCCCCTCCCTGTGCCG	*XbaI* NdeI
bchP^{Bv}DownF	CGGCACAGGGAGGGGATGAGCATATGCATGCA*TCTAGA*CCGATCCGTCGCGGCCGCCTGC	NdeI *XbaI*
bchP^{Bv}DownR	GTCAAGCTTCCGAAGAACGCCAGCACGAACACC	HindIII
bchP^{Bv}aadAF	CATGCATATGGCTTGTTATGACTGTTTTTTTGG	NdeI
bchP^{Bv}aadAR	CAGTCTAGATTATTTGCCGACTACCTTGGTG	XbaI
bchP^{Bv}CheckF	GCCGAGGCCGCTGCCGCCAAG	
bchP^{Bv}CheckR	CAGCCAATGGGTGCCGACAACC	
bchO^{Cpar} pBB F	CGGGATCCATGAAACCGTTTGATGTGGTGATTTCC	BamHI
bchO^{Cpar} pBB R	GCGACTAGTTCAAAACAACGGATTGAGCAGATTCACC	SpeI
bchO^{Ctha} pBB F	CGGGATCCATGCAAACTGAAGTTTTGGTGGTTGG	BamHI
bchO^{Ctha} pBB R	GCGACTAGTTCAAGAAAATTCCACATCATCAAAAAGC	SpeI
bchO^{Paes} pBB F	CGGGATCCATGCAATGCTATGATGCTGTTATTTCGG	BamHI
bchO^{Paes} pBB R	GCGACTAGTTTAAAAAAACGGTATCAGCATTTTCAGCAACTCG	SpeI
bchO^{Ccla} pBB F	CGGGATCCATGAAATCCTATGATGTTGTTATATCAGG	BamHI
bchO^{Ccla} pBB R	GCGACTAGTTCACGAAAAAGGAGGTATTAATTTGAGC	SpeI
bchO^{Gpho} pBB F	ATAGATCTATGGTGTCTTCCGTTCCCGTCC	BglII
bchO^{Gpho} pBB R	GCGACTAGTTCAGGCGAAGAACCGCAGCAGG	SpeI

Table S3. List of primers used in this study.

Supporting Information References

1. Simon, R., Priefer, U., and Pühler, A. (1983) A broad host range mobilization system for *in vivo* genetic engineering: transposon mutagenesis in Gram negative bacteria. *Nat. Biotechnol.* 1, 784-791.
2. Eisen, J. A., Nelson, K. E., Paulsen, I. T., Heidelberg, J. F., Wu, M., Dodson, R. J., Deboy, R., Gwinn, M. L., Nelson, W. C., Haft, D. H., Hickey, E. K., Peterson, J. D., Durkin, A. S., Kolonay, J. L., Yang, F., Holt, I., Umayam, L. A., Mason, T., Brenner, M., Shea, T. P., Parksey, D., Nierman, W. C., Feldblyum, T. V., Hansen, C. L., Craven, M. B., Radune, D., Vamathevan, J., Khouri, H., White, O., Gruber, T. M., Ketchum, K. A., Venter, J. C., Tettelin, H., Bryant, D. A., and Fraser, C. M. (2002) The complete genome sequence of *Chlorobium tepidum* TLS, a photosynthetic, anaerobic, green-sulfur bacterium. *Proc. Natl. Acad. Sci. U.S.A.* 99, 9509-9514.
3. Chi, S. C., Mothersole, D. J., Dilbeck, P., Niedzwiedzki, D. M., Zhang, H., Qian, P., Vasilev, C., Grayson, K. J., Jackson, P. J., Martin, E. C., Li, Y., Holten, D., and Hunter, C. N. 2015. Assembly of functional photosystem complexes in *Rhodobacter sphaeroides* incorporating carotenoids from the spirilloxanthin pathway. *Biochim. Biophys. Acta-Bioenergetics* 1847, 189-201.
4. Ortega-Ramos, M., Canniffe, D. P., Radle, M. I., Hunter, C. N., Bryant, D. A., and Golbeck, J. H. (2018) Engineered biosynthesis of bacteriochlorophyll g_F in *Rhodobacter sphaeroides*. *Biochim. Biophys. Acta-Bioenergetics* 1859, 501-509.
5. Tikh, I. B., Held, M., and Schmidt-Dannert, C. (2014) BioBrick™ compatible vector system for protein expression in *Rhodobacter sphaeroides*. *Appl. Microbiol. Biotechnol.* 98, 3111-3119.
